# The role of faecal calprotectin in diagnosis and staging of colorectal neoplasia: a systematic review and meta-analysis

**DOI:** 10.1186/s12876-022-02220-1

**Published:** 2022-04-09

**Authors:** Fiona A. Ross, James H. Park, David Mansouri, Emilie Combet, Paul G. Horgan, Donald C. McMillan, Campbell S. D. Roxburgh

**Affiliations:** Academic Unit of Surgery, School of Medicine, University of Glasgow, Glasgow Royal Infirmary, Level 2, New Lister Building, 10-16 Alexandra Parade, Glasgow, G31 2ER UK

**Keywords:** Colorectal cancer, Neoplasia, Faecal calprotectin

## Abstract

**Introduction:**

The presence of inflammation is a key hallmark of cancer and, plays an important role in disease progression and survival in colorectal cancer (CRC). Calprotectin detected in the faeces is a sensitive measure of colonic inflammation. The role of FC as a diagnostic test that may categorise patients by risk of neoplasia is poorly defined. This systematic review and meta-analysis aims to characterise the relationship between elevations of FC and colorectal neoplasia.

**Methods:**

A systematic review was performed using the keywords (MESH terms) and a statistical and meta-analysis was performed.

**Results:**

A total of 35 studies are included in this review. CRC patients are more likely than controls to have an elevated FC OR 5.19, 95% CI 3.12–8.62, *p* < 0.001 with a heterogeneity (I^2^ = 27%). No tumour characteristics significantly correlated with FC, only stage of CRC shows signs that it may potentially correlate with FC.

**Conclusion:**

FC levels are significantly higher in CRC, with high sensitivity. Its low specificity prevents it from being used to diagnose or screen for CRC.

**Supplementary Information:**

The online version contains supplementary material available at 10.1186/s12876-022-02220-1.

## Background

Colorectal cancer (CRC) is the fourth most common cancer and second leading cause of cancer death in the United Kingdom (UK) [1]. Overall survival remains poor and across all stages of disease approximately only 50% of patients survive 5 years from presentation [1]. Population-based bowel cancer screening programs have been implemented globally with the aim of clearing premalignant lesions and detecting CRC at an earlier stage to improve overall CRC mortality [2].

The optimal screening technique is not yet known and while current methods including faecal occult blood test (FOBT) and faecal immunochemical testing (FIT) combined with colonoscopy appear sensitive, their specificity is lacking [3]. Colonoscopy is an invasive, expensive test and strategies to improve current diagnostic and screening models would be beneficial. Improving the sensitivity and specificity of non-invasive investigations in diagnosis of CRC is therefore highly sought after.

Colonic inflammation can drive carcinogenesis and the presence of an inflammatory based microenvironment is a key hallmark of cancer [4]. Furthermore, the role of inflammatory responses at a local and systemic level, play important roles in disease progression and survival in CRC [5–7]. Current methods of assessment of local inflammation rely on tissue sampling which may not be appropriate for population-based screening [5, 8, 9].

Calprotectin detected in the faeces is one sensitive measure of colonic inflammation used mainly in the clinical assessment of inflammatory bowel disease (IBD) [10]. It belongs to the S-100 protein family, consisting of three polypeptide chains and is found predominantly in the cytoplasm of neutrophils and the membrane of monocytes [10–12]. Calprotectin is released upon neutrophil cell death or damage [13], and is thought to have regulatory roles on components of the inflammatory process including other myeloid derived cells (e.g. CD11b+ cells) [10, 14].

Calprotectin enters the bowel lumen by migration [15] and is resistant to enzymatic degradation and therefore can be readily detected in bodily fluids such as faeces [11, 14, 16]. Elevation of faecal calprotectin (FC) occur in a wide variety of GI conditions including colitis and malignancy and although a sensitive measure of inflammation, it is not specific for any single condition. Nonetheless, given the importance of inflammation in cancer development and progression, the presence of an elevated FC may provide additional discrimination of a patient’s risk of colorectal neoplasia and progression.

The role of FC as a diagnostic test that may categorise patients by risk of neoplasia (adenomas and carcinomas) is poorly defined. Furthermore, it is not clear whether FC values show any correlation with tumour characteristics including disease stage or location. The aim of this systematic review and meta-analysis is to attempt to characterise the relationship between elevations of FC and colorectal neoplasia, in order to ascertain whether there may be any value in its routine assessment as part of the diagnostic process.

## Methods and materials

A systematic review was performed with the aim primarily to define the relationship between FC and presence of colorectal neoplasia and secondarily whether FC can be used to aid staging of colorectal cancer. Review methodology followed the Preferred Reporting Items for Systematic Reviews and Meta-analyses (PRISMA) statement.

### Eligibility criteria

To be included in this review, the studies had to examine either FC in relation to colorectal neoplasia (including cancer) or in relation to stage of colorectal cancer, in human studies of participants aged > 18 years. Studies looking at the relationship of FC to other pathologies (not including cancer), animal, children, pre-clinical, non-English, duplicates and abstract-only studies were excluded.

### Information sources

A literature search was made of the US National Library of Medicine (MEDLINE, via PubMed), the Cochrane Database of Systematic Reviews and Ovid. Search was performed from inception to 31 March 2017. The search was later extended to 31 March 2021. The bibliographies of relevant studies were hand-searched for any additional relevant studies to be included.

### Search strategy

The following search terms were used “calprotectin AND (neoplasia OR malignancy OR cancer)”. These final search terms were chosen after a number of provisional searches because they returned the greatest number of relevant abstracts to the review topic.

### Selection process

**Fig. 1:**
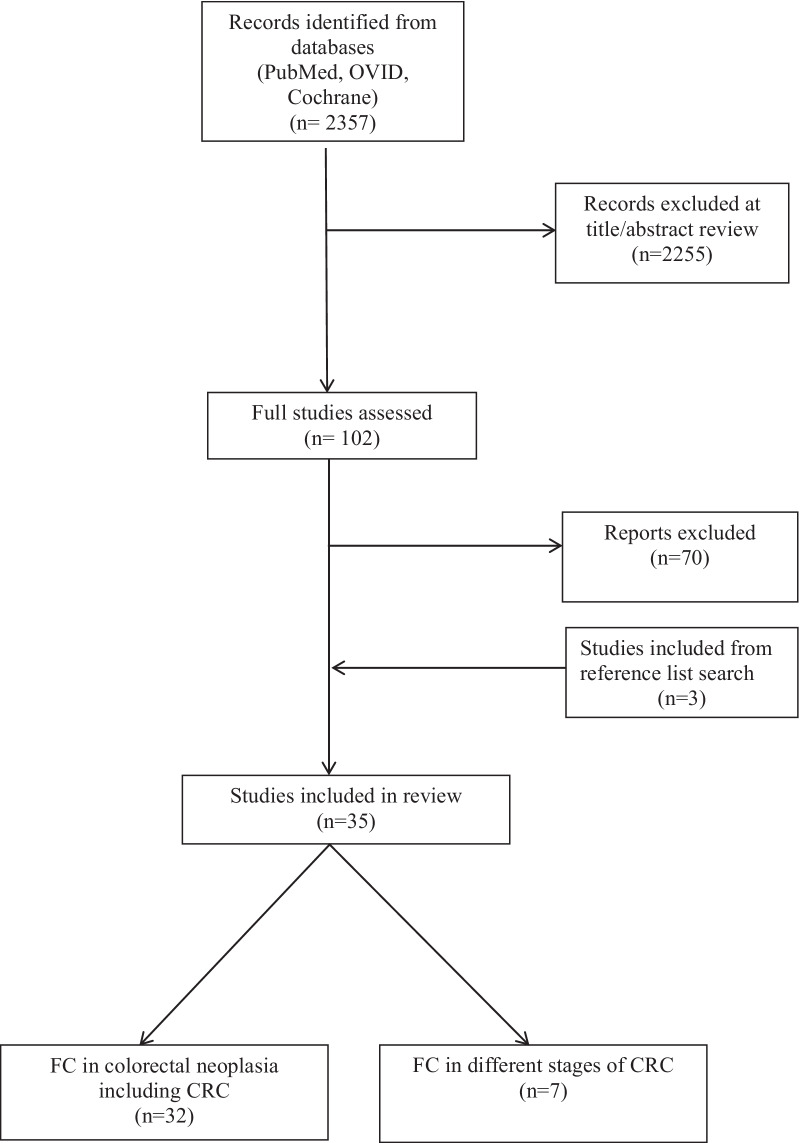
PRISMA flow chart of study selection process

The titles and abstracts of all the studies returned by the search terms were reviewed. The full text of studies not excluded at this stage were obtained and reviewed, to determine if they meet the inclusion and exclusion criteria. The selected were studies were grouped into ‘FC in colorectal neoplasia’ and ‘FC in different stages of CRC’. Four of the included papers investigated both FC in colorectal neoplasia, as well as in different stages of CRC. This selection process was performed by one researcher (FR). This selection process is summarised in the flow chart (Fig. 1).

### Data collection and synthesis

A standardised form was used for recording data extraction and collection for each paper. This encompassed paper details including author and year, whether the paper met the required criteria, sample size, indication for FC in patients, how FC was assessed and what measurement and cut-off of FC was used. Sensitivity and specificity data was retrieved directly from the studies. In some studies the raw data was given, and this enabled this data to be calculated. Meta-analysis (random effect model) of FC levels in colorectal neoplasia (adenomas, advanced adenomas and CRC) was undertaken. Values were again extracted directly from the studies or calculated from the values given.

Data for colorectal neoplasis was assessed in the form of adenomas, advanced adenomas and colorectal cancer. Adenomas were considered advanced adenomas if they were > 10 mm in size, had severe dysplasia or villous components. Adenomas without these features were grouped together and termed low-risk.

Several tests are available in the UK for measuring FC. The Enzyme-linked immunosorbent assay (ELISA), a newer assay with reported as being five times more sensitive in comparison to the original (μg/g rather than mg/L) [17]. Old and new results can be directly compared by simply multiplying the former by a factor of 5 [17]. To allow comparison of the two main units these have all been standardised, for this review, by converting mg/l to μg/g as described above.

### Risk of bias assessment

Bias was recorded as unclear risk for all studies. Overall bias has been illustrated in funnel plots in the Additional file 1: Figures.

### Statistical analysis

Data analysis was performed using Review Manager (RevMan) Version 5.3 (The Nordic Cochrane Centre, The Cochrane Collaboration, Copenhagen, 2014). *p* < 0.05 was considered significant and heterogeneity was assessed with the I^2^ test. Forest plots were created to display the study results, with the overall odds ratio (OR) and 95% confidence interval. Sensitivity and specificity, positive predictive value (PPV) and negative predictive value (NPV) calculations was performed manually.

## Results

### Study selection process

The selection process is summarised in Fig. 1. Using the search protocol described in the methods, 2357 papers were found. The titles and/or abstracts for these were reviewed and 1980 were excluded based on abstract review. Full text analysis was completed for 102 papers and 32 were included for the purposes of this review. The reference lists of the final papers were hand searched and three additional relevant papers were included. Thirty five papers were included in total. Four of the included papers investigated both FC in colorectal neoplasia, as well as in different stages of CRC.

### Units for faecal calprotectin and cut-off points

Several tests are available in the UK for measuring FC. The most common being Enzyme-linked immunosorbent assay (ELISA), which was first used by Roseth et al. [18]. In 2000, a new assay became available which was reported as five times more sensitive in comparison to the original (μg/g rather than mg/L) [17]. Old and new results can be directly compared by simply multiplying the former by a factor of 5 [17]. There is currently no preference for what test should be performed in the UK [19]. The current cut-off for ‘normal’ has been defined by manufacturers at 50 μg/g, with some going further and stating that levels of ≥ 200 μg/g signify that inflammation is present and further investigations are required [20]. Cut-off points for specific colonic diseases including neoplasia are not known.

The studies in this review cover a variety of assays and cut-off points reflecting the long time period of more than 25 years (1992–2021) over which FC has been analysed and the technological advances that have occurred. Table 1 details the testing methods and thresholds for FC.

**Table 1:** Studies reporting faecal calprotectin assays in the context of colorectal neoplasia

References	Year	Manufacturer	Test	Units	Cut-off	Standardised cut-off
Roseth [18]	1992		EIA	μg/l		
Roseth [21]	1993		EIA	mg/l	10 mg/l	50
Gilbert [22]	1996	Nycomed Pharma	ELISA	mg/l	10 mg/l	50
Kristinsson [23]	1998		EIA	mg/l	10 mg/l	50
Kronborg [24]	2000	Nycomed Pharma	ELISA, PhiCal	mg/l	10 mg/l	50
Ton [11]	2000	Nycomed Pharma	ELISA, PhiCal	mg/l	10 mg/l	50
Kristinsson [16]	2001	Nycomed Pharma	ELISA, PhiCal	mg/l	10 mg/l	50
Tibble [25]	2001		ELISA	mg/l	10 mg/l	50
Kristinsson [26]	2001	Nycomed Pharma	ELISA, PhiCal	mg/l	10 mg/l	50
Summerton [27]	2002	Nycomed	ELISA, PhiCal	mg/l	10 mg/l	50
Tibble [28]	2002		ELISA	mg/l	10 mg/l	50
Costa [29]	2003	Eurospital	ELISA, Calprest	μg/g	50 μg/g	50
Limburg [30]	2003	Nycomed Pharma	ELISA	μg/g	50 μg/g	50
Hoff [31]	2004	Nycomed Pharma	ELISA, PhiCal	μg/g	50 μg/g	50
Chung-Faye [32]	2007		ELISA	μg/g	25 μg/g	25
Damms[33]	2008	Bühlmann	ELISA	μg/g	50 μg/g	50
Karl [34]	2008	NovaTec	ELISA	μg/g		
Meucci [35]	2010	Eurospital	ELISA, Calprest	mg/dl	50 mg/dl	
Kalimutho [36]	2011	Eurospital	ELISA, Calprest	ng/ml	45.8 ng/ml	
Kok [37]	2012	Bühlmann	ELISA, EK-CAL	μg/g	50 μg/g	50
Manz [38]	2012	Bühlmann	ELISA	μg/g	50 μg/g	50
Parente [39]	2012	Bühlmann	ELISA	μg/g	50 μg/g	50
Pavlidis [40]	2013	Bühlmann	ELISA, EK-CAL	μg/g	50 μg/g	50
Khoshbaten [41]	2014	Bühlmann	ELISA	μg/g	75.8 μg/g	75.8
Lehmann [42]	2014	Viollier	ELISA	μg/g	50 μg/g	50
Wang [43]	2014	Labsystem	ELISA	IU/ml		
Borza [44]	2015	Sofar Farmaceutici	Cal-Detect SOFAR (Semi-quantitative)	mg/g	15 mg/g	
Mowat [45]	2015	Bühlmann	ELISA, EK-CAL	μg/g	50 μg/g	50
Cubiella [46]	2016	Bühlmann	ELISA, fCAL	ng/ml		
Rutka [47]	2016	Bühlmann	Quantum Blue	μg/g	128.5 μg/g	128.5
Turvill [48]	2016	Bühlmann	ELISA, EK-CAL	μg/g	50 μg/g	50
Widlak [49]	2016	Thermo Fisher Scientific	ELISA, EliA	μg/g	50 μg/g	50
Hogberg [50]	2017	Calpro AS	ELISA, CALPRO	μg/g	100 μg/g	100
Turvill [51]	2018	Bühlmann	ELISA, EK-CAL	μg/g	10 μg/g	10
Lue [52]	2020	Thermo Fisher Scientific	ELISA, EliA	μg/g	50 μg/g	50

*EIA* enzyme immunoassay, *ELISA* enzyme-linked immunosorbent assay, *mg/l* milligrams/litre, *mg/dl* milligrams/ desilitre, *μg/g* microgram/gram, *ng/ml* nanogram/millilitre, *IU/ml* international unit/millilitre, *Standardised Cut-off* μg/g or mg/l multiplied by 5

A full table of all papers and how patients were recruited for each study can be found in the Additional file 1: Tables.

### Faecal calprotectin and adenomas

We hypothesise that FC would be lowest in patients with no colorectal pathology with a sequential rise through the stages of neoplasia.

Nineteen studies examined whether FC correlates with degree of colorectal adenomas. Five of the nineteen studies were not included in the final analysis as insufficient information was available to allow for direct comparison. Nine studies including 5350 patients reported median values for their datasets (Table 2). Twelve studies including 6555 patients reported sensitivity and specificity data (Table 3). Seven studies did not report sensitivity, specificity, PPV or NPV, but reported data that allowed for these to be calculated. Three of the eleven studies [39, 47, 48] did not report specific sensitivity or specificity figures, or allow for these figures to be calculated. However, significance of results was reported therefore the studies have been included in this review.

**Table 2:** Median faecal calprotectin levels in colorectal neoplasia

Author	Year	n					Median			
		Total	Normal (%)	Adenoma (%)	AA (%)	CRC (%)	Normal	Adenoma	AA	CRC
Roseth	1992	111	33 (29.7)	–	–	8 (7.2)	2025 μg/l	–	–	40000 μg/l
Roseth	1993	206	113 (54.8)	–	–	53 (27.7)	–	–	–	50 mg/l
Gilbert	1996	18	4 (22.2)	–	–	14 (77.8)	5 mg/l	–	–	33 mg/l
Kristinsson	1998	119	–	–	–	119 (100)	5.2 mg/l	–	–	50 mg/l
Kronborg	2000	814	488 (60.0)	203 (24.9)	–	23 (2.8)	6.6 mg/l	9.1 mg/l	–	17.6 mg/l
Ton	2000	238	59 (24.8)	–	–	149 (62.6)	26 μg/g	–	–	372 μg/g
Kristinsson	2001	237	114 (48.1)	73 (30.8)	17 (7.2)	5 (2.1)	11.5 mg/l	14 mg/l	–	18 mg/l
Tibble	2001	233	96 (41.2)	29 (12.4)	–	62 (26.6)	2.3 mg/l	12 mg/l	–	101 mg/l
Summerton	2002	134	28 (20.9)	6 (4.5)	–	8 (6.0)	4.5 mg/l	3.8 mg/l	–	53.5 mg/l
Tibble	2002	602	–	–	–	7 (1.2)	–	–	–	47 mg/l
Hoff	2004	2321	1518 (65.4)	592 (25.5)	195 (8.4)	16 (0.7)	21.5 μg/g	–	24 μg/g	66.1 μg/g
Chung-Faye	2007	148	–	–	–	7 (4.7)	15 μg/g	–	–	105 μg/g
Damms	2008	140	56 (40.0)	29 (20.7)	–	8 (5.7)	25.8 μg/g	66.3 μg/g	–	164 μg/g
Karl	2008	551	252 (45.7)	–	113 (20.5)	186 (33.8)	22.4 μg/g	–	27.2 μg/g	420.5 μg/g/ 350.3 μg/g
Kok	2012	382	112 (29.3)	53 (13.9)	16 (4.2)	19 (5.0)	46 μg/g	71 μg/g	89 μg/g	274 μg/g
Manz	2012	538	314 (58.4)	50 (9.3)	–	17 (3.2)	10 μg/g	101 μg/g	–	104 μg/g
Khoshbaten	2014	150	50 (33.3)			50 (33.3)	19.3 μg/g			19.3 μg/g
Lehman	2014	80	–	–	–	80 (100)	–	–	–	205 μg/g
Wang	2014	40	20	–	–	20 (100)	116 IU/ml	–	–	179.1 IU/ml
Turvill	2016	654	–	–	–	39 (6.0)	–	–	–	272 μg/g
Widlak	2016	430	–	42 (9.8)	–	24 (5.6)	–	–	–	145 μg/g
Cubiella	2016	1572	–	–	–	214 (13.6)	–	–	–	120 ng/ml

*CRC* colorectal cancer, *AA* advanced adenoma

**Table 3:** Sensitivity and specificity data for faecal calprotectin in adenomas and advanced adenomas

Author	Year	n			Cut-off	Standard-ised cut-off	Sens (%)	Spec (%)	PPV (%)	NPV (%)	Comment
		Total	Adenoma (%)	AA (%)							
*Adenoma***s**											
Kronborg	2000	814	203 (24.9)	–	10 mg/l	50	43.0	–	–	–	
Tibble	2001	233	29 (12.4)	–	10 mg/l	50	55.0	85.2*	45.7*	89.3*	
Kristinsson	2001	237	73 (30.8)	17 (7.2)	10 mg/l	50	56.2	47.4	40.6*	62.8*	
					15 mg/l	75	45.2	59.6	41.8*	63.0*	
					20 mg/l	100	31.5	71.1	41.1*	61.8*	
Damms	2008	140	29 (20.7)	–	50 μg/g	50	55.0	79.0	57.0	77.0	
Kalimutho	2011	192	69 (35.9)	34 (17.7)	45.8 ng/ml		28.0	25.0*	21.0*	34.0*	
Widlak	2016	430	42 (9.8)	–	50 μg/g	50	43.0	56.0	10.0*	90.0*	
Rutka	2016	95	36 (37.9)	20 (21.1)			–	–	–	–	Faecal calprotectin significantly lower in low-risk adenoma compared to CRC
*Advanced adenomas*											
Hoff	2004	2321	–	195 (8.4)	50 μg/g	50	26.7*	76.1*	12.5*	89.0*	
Mowat	2015	755	–	41 (5.4)	50 μg/g	50	58.5	37.8	5.3	93.8	
					200 μg/g	200	19.5	73.7	4.3	93.8	
Lue	2020	404	41 (10.1)	39 (10)	50 μg/g	50	66.6	48.8	12.2	93.2	
Parente	2012	280	–	85 (30.4)			–	–	–	–	Significant differences between faecal calprotectin in both CRC and AA, and normal and AA (*p* < 0.001)
Turvill	2016	654	–	33 (5.0)			–	–	–	–	30/33 (90.9%) patients with AA had a high faecal calprotectin

*Calculated value, *AA* advanced adenoma, *PPV* positive predictive value, *NPV* negative predictive value, standardised Cut-off: μg/g or mg/l multiplied by 5, –: no information available/ unable to calculate based on available information

Seven out of nine studies showed median FC levels were higher in adenomas and in turn colorectal cancer, in comparison to normal patients. Six out of seven studies reported higher median FC levels specifically in adenomas compared to patients with no colorectal pathology. All nine studies reported lower levels of FC in adenomas or advanced adenomas in comparison to colorectal cancer.

The sensitivity and specificity of FC for both adenomas and advanced adenomas covered a wide range. For adenomas a sensitivity ranged from 28.0 to 56.2% and specificity 25.0–85.0%, and correspondingly in advanced adenomas 26.7–66.6% sensitivity and specificity 37.8–76.1%, using the cut-offs mentioned in the previous section. As the cut-off value for FC increased, the sensitivity for both adenomas and advanced adenomas reduced and specificity increased.

**Fig. 2:**
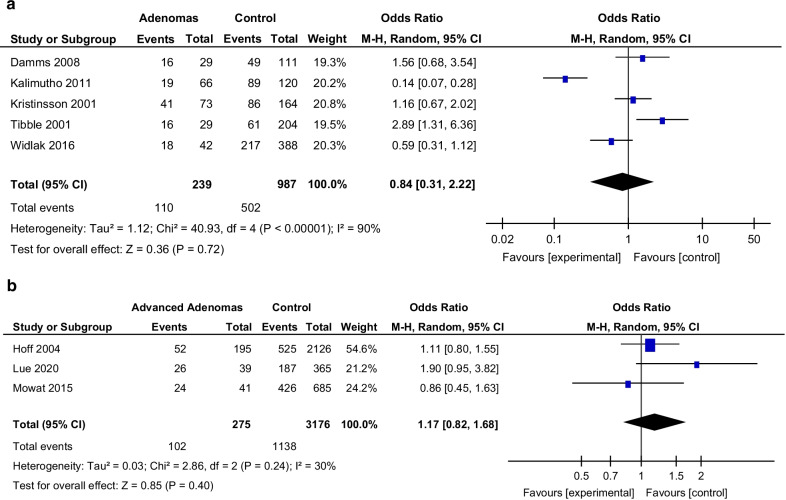
**a** Forest plot—faecal calprotectin in adenoma. **b** Forest plot—faecal calprotectin in advanced adenoma

The PPV is lower than the NPV for all studies for both adenomas and advanced adenomas reflecting the sensitive (but not specific) nature of FC. For advanced adenomas in particular, the NPV was > 89% in all studies and PPV was < 25%. This suggests that in patients with a normal FC, it is less likely that they will have an advanced adenoma, however a high FC does not confer with an adenoma or advanced adenoma specifically. Five studies were included in a meta-analysis of FC levels in patients with adenomas (Fig. 2a). In this small number of studies, OR ranged from 0.13 14 to 2.89, overall OR 0.84 (95% CI 0.31–2.22) with high heterogeneity (I^2^ = 90%), *p* = 0.72. Three studies were included in meta-analysis of FC levels in patients with advanced adenoma (Fig. 2b). This showed overall OR 1.17 (95% CI 0.82–1.68), I^2^ = 30%, *p* = 0.40. This shows a lack of evidence supporting an association between FC and adenoma/advanced adenomas and confirms the variable nature of FC in relation to its use in adenomas.

### Faecal calprotectin and colorectal cancer

Thirty-four studies examined whether FC correlates with CRC. Three of the thirty-four studies were not included in the final analyses as insufficient information was available to allow for direct comparison. Twenty-two studies including 1128 patients with CRC reported median FC values for their datasets in CRC (Table 2). Pavlidis et al. [40] was excluded as it only included one CRC patient. Fifteen studies including 8197 patients (429 CRCs) reported sensitivity and specificity data (Table 4). Borza et al. [53] was excluded from this table, because while they reported sensitivity and specificity, the comparison was cancer of diabetic patients vs cancer of non-diabetic patients. Three studies did not report sensitivity, specificity, PPV or NPV, but reported data that allowed for these to be calculated.

**Table 4:** Sensitivity and specificity data for faecal calprotectin in colorectal cancer

Author	Year	n		Cut-off	Standard-ised cut-off	Sens (%)	Spec (%)	PPV (%)	NPV (%)	Comment
		Total	CRC (%)							
Kronborg	2000	814	23 (2.8)	10 mg/l	50	74.0	–	–	–	
Tibble	2001	233	62 (26.6)	10 mg/l	50	90.0	–	–	–	
Hoff	2004	2321	16 (0.7)	50 μg/g	50	72.7*	76.1*	4.2*	99.5*	
Damms	2008	140	8 (5.7)	50 μg/g	50	100	79.0	40.0	100	
Meucci	2010	870	34 (3.9)	50 mg/dl		85.0	58.0	6.0	99.0	
Kalimutho	2011	192	28 (14.6)	45 .8 ng/ml		72.0	75.0	43.0*	91.0*	
Parente	2012	280	47 (16.8)	50 μg/g	50	85.7	39.7	22.2	93.3	
				416 μg/g	416	43.2	88.8	44.2	88.4	
Khoshbaten	2014	150	50 (33.3)	75.8 μg/g	75.8	80.0	84.0	–	–	
Mowat	2015	755	28 (3.7)	50 μg/g	50	82.1	38.8	5.1	98.2	
				200 μg/g	200	46.4	74.9	6.9	97.2	
Rutka	2016	95	19 (20.0)	128.5 μg/g	128.5	77.8	70.0	53.8	87.5	
Turvill	2016	654	39 (6.0)	50 μg/g	50	92.7	35.2	8.7	98.6	
Widlak	2016	430	24 (5.6)	50 μg/g	50	68.0	84.0	21.0*	98.0*	Total number for this analysis is 25 CRC (including 1 HGD)
Hogberg	2017	373	8 (2.1)	20 μg/g	20	100	51.5	4.3	100	
				50 μg/g	50	87.5	72.1	6.4	99.6	
				100 μg/g	100	50.0	85.2	6.9	98.7	
Turvill	2018	515	27 (5.2)	10 μg/g	10	74.1	66.3	10.9	97.9	For a single FC
Lue	2020	404	16 (4)	50 μg/g	50	75	48.2	5.6	97.9	

*Calculated value, *CRC* colorectal cancer, *PPV* positive predictive value, *NPV* negative predictive value

For healthy individuals, median calprotectin was low with range of 2.3–11.5 mg/l and 10–46 μg/g. In CRC the median calprotectin was higher with range 17.6–101 mg/l and 19.3–420.5 μg/g. Median FC was higher in CRC in fifteen out of the sixteen studies that reported median values for both healthy and CRC subjects (with half of the studies reporting a significant difference). Only one study reported the same results for both healthy individuals and those with cancer [41]. All nine studies reporting median FC in both adenoma and CRC patients reported higher median FC in CRC compared to any degree of adenomas.

Sensitivity and specificity range from 68.0 to 100% and 35.2 to 84.0% respectively in CRC. As the cut-off for FC increases, there is a fall in sensitivity with a corresponding rise in specificity. In CRC FC (using cut off 50 µg/g) has a high negative predictive value with seven out of eight studies reporting a value > 95%, and all studies reporting a NPV > 85%. However, this is at the detriment of a low positive predictive value with five out of eight studies reporting a value < 10%.

**Fig. 3:**
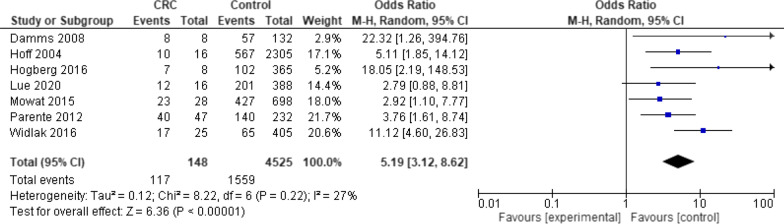
Forest plot—faecal calprotectin in colorectal cancer

Meta-analysis of seven studies of FC in CRC was performed (Fig. 3). Patients with CRC are fivefold more likely than controls to have an elevated FC (OR 5.19, 95% CI 3.12–8.62, *p* < 0.001 with a heterogeneity (I^2^ = 27%)).

### Faecal calprotectin and staging of colorectal cancer

There are fewer studies reporting FC’s relationship to stage of disease or tumour histopathology, in colorectal cancer. In this review eight studies incorporated various elements of this relationship.

Seven of these studies report on FC in different stages of colorectal cancer. For comparison these have been grouped per stage of disease (Table 5). Lehman et al. [42] showed that T-stage correlated with FC with patients with T3/4 disease having significantly higher FC levels than T1/2 disease (*p* = 0.022). Kristinsson et al. [16] reported that those with Dukes A disease had lower FC levels, but this result was not of statistical significance. This is similar to that reported by Karl et al. [34] with median FC levels of 179.2 μg/g in Dukes A and at least > 300 μg/g for Dukes B–D. However no other study has shown any significant correlation between FC and stage of colorectal cancer.

**Table 5:** Faecal calprotectin levels in different stages of colorectal cancer

Author	Year	CRC total n	Stage				Comment
			0/I	II	III	IV	
			n (%)	n (%)	n (%)	n (%)	
			FC (Median)	FC (Median)	FC (Median)	FC (Median)	
Gilbert	1996	14		5 (36.0)	2 (14.0)	7 (50.0)	Stage had no effect on faecal calprotectin levels
Kristinsson	1998	119	25 (21.0)	33 (28.0)	36 (30.0)	25 (21.0)	No significant difference
			50 mg/l	65 mg/l	34 mg/l	38 mg/l	
Kristinsson	2001	155	20 (13.0)	66 (43.0)	45 (29.0)	23 (15.0)	No significant difference
			27 mg/l	49 mg/l	42 mg/l	48 mg/l	
Tibble	2001	62	10 (16.0)	24 (39.0)	14 (23.0)	14 (23.0)	No significant difference (*p* > 0.2)
			62.5 mg/l	115 mg/l	62 mg/l	132 mg/l	
Karl	2008	85 (186)	23 (27.0)	27 (32.0)	12 (14.0)	23 (27.0)	85/186 CRC had stage specified
			179.2 μg/g	550.2 μg/g	542.5 μg/g	312.8 μg/g	
Kalimutho	2011	28	7	5	3		18 CRC either did not have FC or stage data
			4/7+ve FC	3/5+ve FC	3/3+ve FC		
Lehman	2014	80					Patients with T3/4 disease had significantly higher FC than T1/2 (*p* = 0.022)

*CRC* colorectal cancer, *FC* faecal calprotectin

Six studies examined whether location of colorectal cancer correlates with FC. Three of these studies looked at the difference between the right and left (but did not specify the exact definition of this). None of the studies showed any significant difference in FC based on the location of the tumour. One study looking only at colorectal neoplasia showed that patients with proximal colonic neoplasms (median 53.8 μg/g) had a higher FC than those with distal neoplasms (median 23.0 μg/g) (*p* = 0.001), however it was based on a small number of cases (16 proximal, 27 distal and 54 both) [30].

One study reported no significant difference in FC between differentiation of disease [23]. Kristinsson et al. [16] reported no significant difference in FC in grade or size of colorectal cancer.

## Discussion

This systematic review and meta-analysis sought to characterise the relationship between elevations of FC and colorectal neoplasia, in order to ascertain whether there may be any value in its routine measurement as part of the diagnostic or pre-operative staging process in CRC.

Ye et al. [54] published a meta-analysis on the diagnostic accuracy of FC for screening for CRC, which reported that FC cannot be recommended for CRC detection. Our current review adds to this literature by aiming to define the relationship between FC and presence of colorectal neoplasia as well as how FC varies with different stages of colorectal cancer.

The potential relationship between FC and colorectal cancer has been of interest since the early 1990s when Roseth et al. [18] first published their work on the subject. The first study was primarily looking at extraction and quantification of calprotectin but found that 10 out of 11 patients with gastrointestinal (GI) cancers had an elevated FC [18]. This was followed up by a pilot study which showed that 94% of CRC patients had elevated FC levels, and a median significantly higher than that of the control group (*p* < 0.0001) [21].

This present study reports that, in adenomas, the relationship with FC has a high degree of variability. The majority of the included studies reported that patients with adenomas had a higher FC than healthy individuals, but lower than those with colorectal cancer. However, the specificity, that would allow FC to be used to diagnose adenomas or differentiate adenomas from other organic pathologies, is absent. Size, location or number of adenomas does not appear to significantly affect FC levels [25, 26].

However, there is a confirmed stronger relationship between FC and CRC. FC is higher in patients with CRC in comparison to both healthy patients and other degrees of neoplasia and is therefore a sensitive marker for CRC. The current globally used standard CRC screening test is FIT, which is moderately sensitive but highly specific for CRC [55]. In FC, the inverse relationship between sensitivity and specificity concedes a low specificity for CRC. This low specificity has prevented FC from becoming a useful screening tool for diagnosing CRC; however it could potentially be used as an adjunct in screening high risk populations [24, 29].

There are also many confounding factors which elevate FC levels including use of common drugs such as proton pump inhibitors (PPIs) and non-steroidal anti-inflammatory drugs (NSAIDs) [56, 57]. In these studies, FC was not found to be influenced by smoking nor alcohol [24, 26]. Kronborg et al. [24] found that diverticulosis increased FC, but not more than polyps without neoplasia, however this was later contradicted when diverticulosis was not found to influence FC levels [26]. These confounding factors decrease FC use as a screening tool for CRC.

In this analysis, each study’s own FC values and ranges, predominantly 10 mg/l or 50 μg/g, were used as a reasonable cut-off point between normal and colorectal pathologies. Patients with a negative FC would be considered low-risk for CRC. Mowat et al. [45] found that a cut-off < 50 μg/g was sufficient to rule out IBD, but missed 5/28 CRC’s and 17/41 higher risk adenomas. In contrast other studies found a higher FC level was a more optimal cut-off point for distinguishing between CRC and normal [34]. Therefore it is unclear what the appropriate cut-off value would be to determine that CRC could be safely excluded based on FC alone. However it would appear that due to the variability and low specificity of FC in CRC that this cut-off value would be too low to be of any useful clinical or financial benefit.

Overall it is widely accepted that FC alone would be a poor screening test for both adenomas and CRC. However it may have a role in clinical diagnosis and staging. Particularly as an additional diagnostic tool to rationalise use of colonoscopy (a timely, expensive and invasive test), to improve risk stratification of both symptomatic and asymptomatic patients.

Many studies have shown a significant fall in FC levels post cancer resection. Kristinsson et al. [23] found that median FC fell significantly from 75 to 10.3 mg/l, after resection. This has again been shown by Kristinsson et al. [16], Lehman et al. [42], and Borza et al. [44]. Despite the lack of evidence supporting the use of FC in screening, this is evidence to show that FC is related to intraluminal tumour burden, and hence may be relevant to clinical diagnosis, pre-operative CRC staging and cancer follow-up. With the exception of Kronborg et all, who reported no significant change in calprotectin levels for all adenoma patients before and after polypectomy, similar data was not available for adenomas post removal in this literature review. Given the low rate of adenoma detection it would be unlikely for FC levels to change significantly following removal, and therefore unlikely for any further role for FC in adenomas.

We therefore hypothesise that patients with a larger intraluminal tumour burden should have higher FC levels. In this analysis we only found one study that reported T-stage significantly (*p* = 0.022) correlating with FC [42], and two further studies showed a non-significant correlation [16, 34]. However no other study showed any significant correlation between FC and stage of CRC. If you consider intraluminal tumour burden as size of tumour rather than depth i.e. T-stage, there was only one study in this review which assessed tumour size in this manner, and there was no correlation found [16]. Therefore more work is required to analyse whether larger or more advanced tumour are expressed by greater FC levels, and can therefore be used in pre-operative CRC staging.

The utilisation of FC in pre-operative staging of CRC is an interesting and novel role. The local inflammatory response also plays an important role in staging and therefore disease progression and survival in CRC [5–7]. Given that FC is a measure of colonic inflammation it may reflect the local inflammatory response, and therefore may be another area where FC can aide staging. As stated previously the current methods of assessing of local inflammation rely on tissue sampling, and assessment of local inflammation is normally post-operative and at present not part of current CRC staging. If using a simple stool sample, the presence of an elevated FC can help discriminate the patients at risk of more advanced disease, and this could potentially be a quick and simple method of advancing pre-operative CRC staging.

However it is not yet clear whether FC correlates with tumour inflammation or histopathology. In ulcerative colitis (UC) it has been shown that disease activity, FC and histology all correlate [58]. Lehman et al. [42] carried out the first study assessing correlation of tumour and histopathological parameters of local inflammation in colorectal cancer. FC did not correlate with any of the markers of local tumour inflammation (Klintrup–Mäkinen grade, lymphocytes, neutrophils, CD3, CD4, CD8, CD45, TIA-1, granzyme B and myeloperoxidase). Kristinsson et al. [23] found no significant correlation in colorectal cancer between FC and markers of systemic inflammation (c-reactive protein (CRP), carcinoembryonic antigen (CEA), plasma calprotectin). More work needs to be performed assessing whether FC in CRC correlates with either the systemic or local inflammatory response.

The limitations of this study are the paucity and heterogeneity of data in the papers. There is variation in the patients, countries, FC assays and cut-offs and other confounding factors. In addition the use of the random effects model means more weight is given to the smaller studies, potentially increasing bias from these smaller and potentially underpowered studies. It is difficult to account for all of these factors, but this heterogeneity itself is the reason why more data and comparative reviews are required to assess whether there is an all-encompassing conclusion.

In conclusion, based on the evidence presented from a range of heterogeneous studies, FC alone would be a poor screening test for colorectal neoplasia, particularly adenomas due to its poor specificity. Further work is required to evaluate whether FC could be used as an adjunct to existing screening methods including FOBT/ quantitative faecal immunochemical test (Q-fit), and whether it could enable rationalisation of colonoscopy in asymptomatic screened patients.

In CRC, the lack of specificity also means that FC would also be a poor cancer screening test. However the high sensitivity of FC in CRC suggests a potential role for FC in the investigation and initial evaluation of CRC. Inflammation is a hallmark of cancer and the role of FC as a diagnostic or prognostic measure in CRC is not defined. In addition, it is unclear whether FC measurement is associated with existing inflammation based assessments in cancer (local and systemic) which if proven may provide a novel role for FC assessment in CRC.

## Supplementary Information

**Additional file 1**. Further data on faecal calprotectin and colorectal neoplasia.

## Data Availability

All data generated or analysed during this study are included in this published article.

## Abbreviations

AA: Advanced adenoma
C: Controls
CEA: Carcinoembryonic antigen
CRC: Colorectal cancer
CRP: C-reactive protein
dx: Diagnosis
EIA: Enzyme immunoassay
ELISA: Enzyme-linked immunosorbent assay
FC: Faecal calprotectin
FH: Family history
FIT: Faecal immunochemical testing
FOBT: Faecal occult blood test
GI: Gastrointestinal
GP: General practitioner
HRA: High risk adenomas
IBD: Inflammatory bowel disease
IDA: Iron deficiency anaemia
NSAIDs: Non-steroidal anti-inflammatory drugs
NPV: Negative predictive value
OGD: Oesophago-gastroduodenoscopy
OR: Odds ratio
PPI: Proton pump inhibitor
PPV: Positive predictive value
Q-fit: Quantitative faecal immunochemical test
SC: Screening
SP: Specific
SY: Symptomatic
T2DM: Type 2 diabetes mellitus
UC: Ulcerative colitis
UK: United Kingdom
