# Supplementary material for: The role of faecal calprotectin in diagnosis and staging of colorectal neoplasia: a systematic review and meta-analysis
Source: BMC Gastroenterol. 2022 Apr 9;22:176. doi: 10.1186/s12876-022-02220-1 (PMC8994317; doi:10.1186/s12876-022-02220-1)
Supplement: Supplementary file 1 — Additional file 1. Further data on faecal calprotectin and colorectal neoplasia. [file 12876_2022_2220_MOESM1_ESM.docx]

### Additional file 1: Tables

| **Patient recruitment to individual trials** | | | | | | | |
| --- | --- | --- | --- | --- | --- | --- | --- |
| **Author** | **Year** | **n** | **Recruitment** | | **Why FC performed?** | **How was CRC diagnosed?** | **IBD excluded?** |
| Roseth | 1992 | 111 | SP/C | Disease specific and controls | Presents new methods for extraction and assessment of FC, and assesses preliminary reference ranges | Pre study | No |
| Roseth | 1993 | 206 | SP/C | Disease specific and controls | Assess whether FC is useful in CRC diagnosis | Pre study | Unknown - not mentioned |
| Gilbert | 1996 | 18 | SP/C | Disease specific and controls | Assess FC in CRC, in comparison to faecal haemoglobin | Pre study Colonoscopy and histology | Unknown – not mentioned |
| Kristinsson | 1998 | 119 | SP | New dx CRC | Assessment of FC in CRC | Pre study | Unknown - not mentioned |
| Kronborg | 2000 | 814 | SC | Screening – high risk individuals | Assess FC for detection of adenomas in high risk individuals | Colonoscopy and histology | No |
| Ton | 2000 | 238 | SP/C | Disease specific and controls | Comparing new and original method of FC analysis | Not mentioned | Unknown – not mentioned |
| Kristinsson | 2001 | 155 | SP | Consecutive new CRC dx | Assess FC levels in pre and post-op CRC resections, and compare to tumour characteristics | Colonoscopy and histology Barium enema | Yes |
| Tibble | 2001 | 233 | SY/C | Consecutive referrals to colonoscopy and controls | Compare FC and FOBT in patients with CRC and polyps to assess use as biochemical marker | Colonoscopy and histology | No |
| Kristinsson | 2001 | 237 | SC | First degree relatives of patients with CRC | Assess whether FC is more sensitive than FOBT in detecting colorectal neoplasia | Colonoscopy and histology | Yes |
| Summerton | 2002 | 134 | SY | Patients with GI sign/ symptoms referred for OGD or colonoscopy | Assess FC as a method of screening for alimentary inflammation and neoplasia | Colonoscopy and histology | No |
| Tibble | 2002 | 602 | SY | Consecutive patients referred to GI clinic | Assess markers of inflammation to distinguish organic from nonorganic intestinal disease | Barium imaging and/or colonoscopy | Yes |
| Costa | 2003 | 239 | SY/C | Consecutive patients referred to clinic and controls | Assessing FC in organic and functional bowel disorders | Colonoscopy and histology Imaging | No |
| Limburg | 2003 | 412 | SY/SC | Referrals to colonoscopy with hx of CR neoplasia, FH CRC or IDA | Assessed FC as a screening biomarker for colorectal neoplasia | Colonoscopy and histology | Unknown – not mentioned |
| Hoff | 2004 | 2321 | SC | Random invitation to population for colorectal examination screening | Assessing non-invasive tests for bowel screening – comparing FC with FOBT | Colonoscopy and histology | Yes |
| Chung-Faye | 2007 | 148 | SY | Clinic attendance with new lower GI symptoms or known IBD | Assessing usefulness of surrogate markers of bowel inflammation | Colonoscopy and histology | No |
| Damms | 2008 | 140 | SY | Referrals to colonoscopy | Assess new rapid FC test, and assess FC potential for use in screening for intestinal inflammation or CRC. | Colonoscopy and histology | No |
| Karl | 2008 | 551 | SY/SP | Referrals to colonoscopy with GI symptoms and specific CRC recruitment | Search for novel biomarkers to improve sensitivity of CRC detection in stool samples | Colonoscopy and histology | Yes |
| Meucci | 2010 | 870 | SY | Outpatient referrals for colonoscopy | Evaluate the role of FC in patients referred for colonoscopy | Colonoscopy and histology | No |
| Kalimutho | 2011 | 192 | SY/SP | Consecutive patients attending for colonoscopy | Compare faecal based DNA integrity to FOBT and FC, for CRC and adenoma detection | Colonoscopy and histology | Yes |
| Kok | 2012 | 382 | SY | Patients attending with persistent lower abdominal complaints | Assess diagnostic accuracy of point of care FC and iFOBT, in suspected organic bowel disease | Colonoscopy and histology | No |
| Manz | 2012 | 538 | SY | Patients attending for endoscopy with abdominal discomfort | Evaluate diagnostic value of FC in patients with abdominal discomfort | Colonoscopy and histology | Unknown – not mentioned |
| Parente | 2012 | 280 | SY | Patients attending GI clinic with abdominal symptoms | Assessed different faecal tests as markers for advanced neoplasia | Colonoscopy and histology | Yes |
| Pavlidis | 2013 | 962 | SY | Patients presenting to GP with persistent GI symptoms | Assess diagnostic performance of FC in routine general practice of symptomatic patients | Colonoscopy and histology Imaging | Yes |
| Khoshbaten | 2014 | 150 | SP/C | Disease specific and controls | Evaluate FC as screening marker for GI malignancy | Colonoscopy and histology | Yes |
| Lehmann | 2014 | 80 | SP | Known CRC, admitted for treatment | Assess FC in pre and post-op CRC resections, and compare to histology | Pre study | Unknown - not mentioned |
| Wang | 2014 | 40 | SP/C | Known CRC and controls | Development and testing of a faecal protein biochip for the screening of CRC | Colonoscopy and histology | Yes |
| Borza | 2015 | 40 | SP | Known CRC (20 with T2DM and 20 without DM) | Assess FC in patients undergoing colorectal cancer surgery, comparatively in patients with and without diabetes | Pre study | Unknown - not mentioned |
| Mowat | 2015 | 755 | SY | Referrals with bowel symptoms to secondary care | Diagnostic accuracy of faecal haemoglobin and FC in symptomatic patients | Colonoscopy and histology | No |
| Cubiella | 2016 | 1572 | SY | Consecutive patients with GI symptoms referred for colonoscopy | To try and develop a CRC predictive model, for symptomatic patients | Colonoscopy and histology | No |
| Rutka | 2016 | 95 | SY | Referrals for colonoscopy | Compare different faecal markers in diagnosis of colorectal adenomas and cancer | Colonoscopy and histology | Unknown – not mentioned |
| Turvill | 2016 | 654 | SY | “2 week wait” referrals for suspected CRC | Determine diagnostic accuracy of FC in patients referred with suspected CRC | Colonoscopy and histology Imaging | No |
| Widlak | 2016 | 430 | SY | Referrals for urgent lower gastrointestinal investigations | Assess FC and FIT in detection of CRC and adenoma in symptomatic patients | Colonoscopy and histology Imaging | No |
| Hogberg | 2017 | 373 | SY | Consecutive patients receiving a FIT or FC test | Assess FC and FIT in detecting CRC, HRA and IBD in primary care | Colonoscopy and histology Imaging | No |
| Turvill | 2018 | 515 | SY | Patients referred for colonoscopy from ‘2 week wait’ colorectal clinics | Diagnostic accuracy in suspected CRC, comparison with FIT | Colonoscopy and histology | No |
| Lue | 2020 | 404 | SY | Symptomatic patients referred for colonoscopy | Diagnostic accuracy and cost-effectiveness of combination of FOBT and FC | Colonoscopy and histology | No |

Additional file 1: Table. Patient recruitment to individual studies

CRC: colorectal cancer, dx: diagnosis, GI: gastrointestinal, OGD: oesophago-gastroduodenoscopy, FH: family history, IDA: iron deficiency anaemia, IBD: inflammatory bowel disease, GP: general practitioner, T2DM: type 2 diabetes mellitus, FIT: faecal immunochemical test, FC: faecal calprotectin, HRA: high risk adenomas SY: Symptomatic, SC: Screening, SP: Specific, C: Controls

| **Faecal calprotectin levels in different locations of colorectal cancer** | | | | | | | |
| --- | --- | --- | --- | --- | --- | --- | --- |
| **Author** | **Year** | **CRC Location** | | | | | **Comment** |
|  |  | **CRC**  Total  n | **Colon**  n (%)  FC (Median) | **Rectum**  n (%)  FC (Median) | **Left**  n (%)  FC (Median) | **Right**  n (%)  FC (Median) |  |
| Gilbert | 1996 | 14 | 13 (93.0) | 1 (7.0) | 79.3 mg/l (mean) | 55.1 mg/l (mean) | No significant difference  (*p*=0.4) |
| Kristinsson | 1998 | 119 | 81 (68.0)  50.0 mg/l | 38 (32.0)  54.5 mg/l | 73 (61.0)  77.4 mg/l | 46 (39.0)  61.6 mg/l | No significant difference |
| Kristinsson | 2001 | 155 | 106 (68.0)  41.5 mg/l | 49 (32.0)  53 mg/l |  |  | No significant difference |
| Tibble | 2001 | 62 | 31 (50.0) | 31 (50.0) |  |  | No significant difference  (*p*>0.5) |
| Limburg | 2003 |  |  |  |  |  | Patients with proximal colonic neoplasms had a higher median FC than distal (Proximal 53.8μg/g, distal 23.0 μg/g *p*=0.001) |
| Lehman | 2014 | 80 |  |  |  |  | No significant difference |
| Widlak | 2016 | 24 |  |  | 143 μg/g | 175 μg/g | No significant difference  (*p*=0.7068) |

Additional file 1: Table. Faecal calprotectin levels in different locations of colorectal cancer

CRC: colorectal cancer, FC: faecal calprotectin


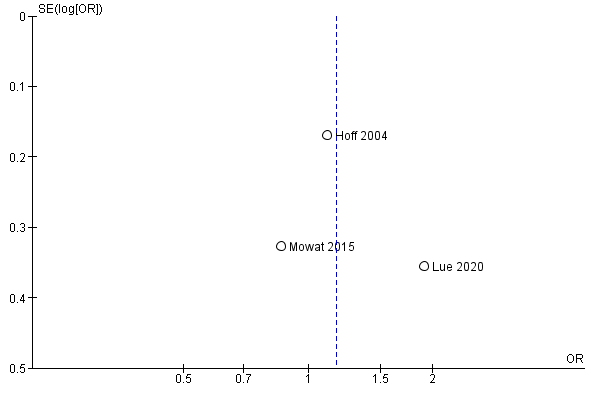


Additional file 1: Figure. Funnel plot of comparison: Faecal Calprotectin in Advanced Adenomas


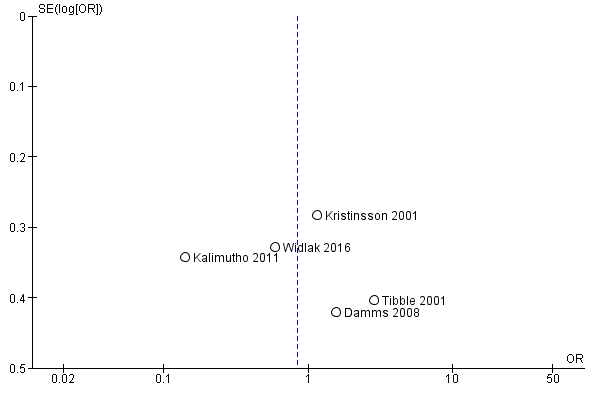


Additional file 1: Figure. Funnel plot of comparison: Faecal Calprotectin and Adenomas


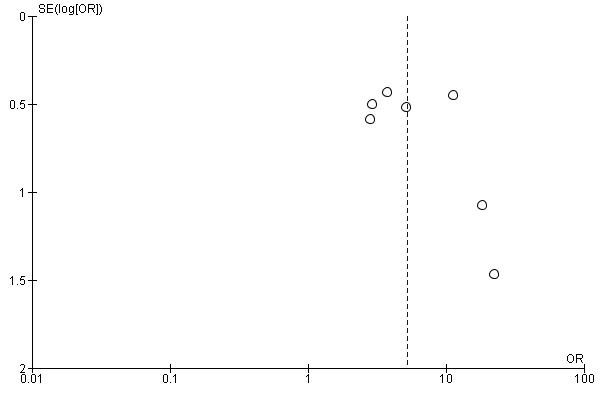


Additional file 1: Figure. Funnel plot of comparison: Faecal Calprotectin and CRC
